# Heterogeneous nuclear ribonucleoprotein K promotes the progression of lung cancer by inhibiting the p53‐dependent signaling pathway

**DOI:** 10.1111/1759-7714.14387

**Published:** 2022-03-29

**Authors:** Mengyuan Li, Xingjiu Yang, Guoxin Zhang, Le Wang, Ziwei Zhu, Wenlong Zhang, Hao Huang, Ran Gao

**Affiliations:** ^1^ National Human Diseases Animal Model Resource Center, The Institute of Laboratory Animal Science Chinese Academy of Medical Sciences & Peking Union Medical College Beijing China; ^2^ NHC Key Laboratory of Human Disease Comparative Medicine Beijing Engineering Research Center for Experimental Animal Models of Human Critical Diseases Beijing China; ^3^ Beijing Engineering Research Center for Experimental Animal Models of Human Critical Diseases Beijing China

**Keywords:** DNA damage response, hnRNPK, lung cancer, p53

## Abstract

**Background:**

Heterogeneous nuclear ribonucleoprotein K (hnRNPK) is a nucleic acid‐binding protein. Reportedly, hnRNPK is overexpressed in many human tumors, and such overexpression is associated with poor prognosis, implicating the role of hnRNPK as an oncogene during tumorigenesis. In this study, hnRNPK expression in lung cancer tissues was investigated.

**Methods:**

Briefly, hnRNPK was knocked down in lung cancer cell lines, and effects of knockdown on the cell proliferation, migration, and cell cycle were assessed using a cell counting kit‐8 (CCK‐8) assay, colony formation assay, transwell assay and flow cytometry. The effects of hnRNPK knockdown on the p53‐dependent signaling pathway were examined using western blotting. Finally, the effect of hnRNPK knockdown on tumor growth was verified in vivo using a lung cancer xenograft mouse model.

**Results:**

hnRNPK knockdown inhibited the cell proliferation, migration and cell cycle. In addition to phenotypic changes, hnRNPK knockdown upregulated expressions of pCHK1, pCHK2, and p53，p21，cyclin D1, thereby mediating the DNA damage response (DDR). The regulatory function of hnRNPK during p53/p21/cyclin D1 signaling in hnRNPK‐knockdown A549 cells was confirmed by suppressed the protein expression of associated signaling pathways, which inhibited DDR.

**Conclusion:**

hnRNPK plays a crucial role in the progression of lung cancer, ultimately affecting survival rate. Inhibition of progression of lung cancer cells induced by hnRNPK‐knockdown is dependent on activation of p53 by the p53/p21/cyclin D1 pathway.

## Graphical abstract

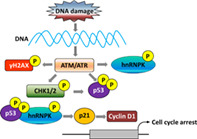

## INTRODUCTION

Heterogeneous nuclear ribonucleoprotein K (hnRNPK) was discovered as a component of the hnRNP complex. hnRNPK preferentially binds to poly(C) and regulates transcription, translation, precursor mRNA splicing, RNA stability, chromatin remodeling, and signal transduction. Several studies have demonstrated that hnRNPK can regulate tumorigenesis and tumor suppressor pathways, overexpression, and knockdown expression. Moreover, it may play roles in cell proliferation and migration inhibition. However, based on clinical data, opinions regarding functions of hnRNPK vary across reports. Reportedly hnRNPK serves as an oncogene in colorectal cancer, nasopharyngeal carcinoma, prostate cancer, melanoma, oral squamous cell carcinoma, and gastric cancer, such that its overexpression is negatively correlated with tumorigenesis and prognosis.^1^, ^2^, ^3^ On the contrary, hnRNPK serves as a tumor suppressor gene in acute myeloid leukemia (AML), as evidenced by the susceptibility of hnRNPK heterozygous mice to AML and lymphoma.^4^ Taken together, although hnRNPK evidently plays an important role in cancer development, its function as an oncogene or a tumor suppressor gene remains debatable.

Mediation of eukaryotic DNA damage response (DDR) can lead to increased cell survival following genomic damage. Ataxia telangiectasia mutated (ATM), ATM‐ and Rad3‐related (ATR), and DNA‐dependent protein kinase catalytic subunit (DNA‐PKcs) are the key DDR regulators, which are activated following DNA damage and phosphorylate downstream targets.^5^ p53 is an important target of ATM and ATR, and it is the most common mutein in human cancers. Loss of p53 function leads to genomic instability and oncogenic mutations that contribute to tumorigenesis.^6^ DNA damage and various other cellular stressors induce significantly increased p53 protein expression. Under normal conditions, p53 activity is low. hDM2 acts as a ubiquitin E3 ligase that binds to and masks the N‐terminal transactivation domain of p53, thereby promoting its degradation via the ubiquitin proteasome pathway.^7^

In the present study, we focused on hnRNPK expression in lung cancer and explored the hypothesis that hnRNPK promotes tumor progression. hnRNPK was generally high in lung cancer tissues, suggesting its oncogenic role. hnRNPK was knocked down in lung cancer cell lines, which resulted in the inhibition of tumor cell proliferation and migration in a p53‐dependent manner. Moreover, further research into the molecular mechanisms underlying this inhibition revealed that hnRNPK knockdown in lung cancer induced DNA damage and activated DDR, which in turn activated p53‐dependent inhibition of the progression of lung cancer.

## METHODS

### Cell lines and cultures

Lung cancer cell lines A549 and H1299 were obtained from the Institute of Basic Medical Sciences, Chinese Academy of Medical Sciences, and cultured in RPMI‐1640 medium (Gibco) containing 10% fetal bovine serum (Gibco), 100 U/ml penicillin, and 100 mg/ml streptomycin (Invitrogen) in a humidified atmosphere of 5% CO_2_ at 37°C.

### Cell transfection

Small interfering RNA (siRNA) against hnRNPK (si‐hnRNPK) and nonspecific control siRNA (si‐NC) were purchased from Ribo (Guangzhou, China). The target sequence of siRNA‐hnRNPK was 5′‐GAGCUUCGAUCAAAAUUGATT and si‐NC was 5′‐UUCUCCGAACGUGUCACGUTT. All cells were transfected with lipofectamine 3000 (Invitrogen) and harvested after 48 h for further analysis.

### Western blotting

Forty‐eight hours after transfection, cells were induced by 500 μM H_2_O_2_ for 24 h. Then, all cells were homogenized in RIPA lysis buffer (Promega) on ice for 30 min. Protein concentrations were estimated using the BCA protein assay (Thermo). Equal amounts of cell lysates were loaded and separated on 12% SDS‐PAGE gels, and proteins were electrotransferred to nitrocellulose membranes, as previously described. The membranes were incubated with a 1:1000 dilution of anti‐hnRNPK, anti‐p53, anti‐p21, anti‐γH2A.X, anti‐pCHK1, anti‐pCHK2, anti‐cyclin D1(Cell Signal Technology), and anti‐β‐actin (1:10000 dilution, Santa Cruz, CA) antibodies overnight at 4°C in 5% nonfat milk, followed by washing three times with TBS‐T buffer and incubation with horseradish peroxidase‐conjugated goat anti‐rabbit IgG (1:10000 dilution with 5% nonfat milk) for 45 min at room temperature. After washing three times with TBS‐T buffer, membranes were visualized using the ECL detection system (Thermo Fisher Scientific).

### Microarray analysis and quantitative real‐time PCR assay

Total RNA was extracted from cultured cells using Trizol reagent (Invitrogen) following the manufacturer's protocol. Gene expression profiles were examined by ShangHai OE Biotechnology Corporation using Agilent SurePrint G3 Human Gene Expression v2 (8 × 60 K, Design ID: 039494) microarrays. After robust multiarray average normalization, fold change thresholds ≥2 and *p*‐values <0.05 were considered to indicate statistically significant alterations. Hierarchical clustering and heat map generation were performed using GeneSpring GX software (Agilent Technologies). Signaling pathways enriched in downstream target genes were generated using the KEGG pathways program (http://www.kegg.jp/).

Complementary DNA (cDNA) was synthesized from 1 μg RNA using ImProm‐II Reverse Transcription System (Promega). Quantitative PCR was performed using the ABI StepOne Real‐Time PCR system (Applied Biosystems) and SYBR Green Real‐Time PCR Master Mix (Toyobo). Primers used for quantitative real‐time PCR are listed in Table S1. All reactions were carried out in triplicates, and relative gene expression was calculated using the comparative cycle threshold (2^−ΔΔCt^) method following the manufacturer's instructions. The house‐keeping gene glyceraldehyde‐3‐phosphate dehydrogenase (GAPDH) was used as an endogenous control to normalize the data. Primers were synthesized by Sangon Biotech.

### Cell proliferation assay

CCK‐8 (Dojindo) was used to evaluate cell proliferation. Cells were seeded into 96‐well plates at a density of 2 × 10^3^ cells per well, followed by the addition of CCK‐8 reagents at different time points and incubation for 3 h at 37°C. Each concentration was set in triplicate. Absorbance was measured using a microplate reader (Bio‐Rad) at a wavelength of 450 nm (OD_450nm_) after slight oscillation for 10 s.

### Colony formation assay

A549 and H1299 cells were seeded into 6‐well culture plates (Corning Inc.) at a density of 1000 cells/well, with each condition set up in triplicate wells. The cells were cultured for 10 days at 37°C constant temperature CO_2_ incubator to induce colony formation (>50 cells per colony). Following the incubation, the cells were fixed with 4% paraformaldehyde at room temperature for 30 min, and then stained with 1% crystal violet at room temperature for 30 min. The colonies were counted and images were captured using a Zeiss Axio Imager Z2 light microscope.

### Transwell assay

A549 and H1299 cells were transfected with si‐hnRNPK and si‐NC as described above. A total of 5 × 10^4^ cells/well were resuspended in 200 μl 1640 without FBS and seeded into the upper compartments of Boyden chambers (Falcon; Corning Inc.) The lower chamber was filled with 600 μl 1640 containing 10% FBS. Following 12 h of incubation at 37°C, the non‐migratory cells remaining in the upper chamber were removed with cotton swabs and the migratory cells in the lower chamber were washed with PBS three times, fixed with 4% paraformaldehyde at room temperature for 30 min and stained with 1% crystal violet for 30 min at room temperature. The stained cells were visualized in five randomly selected fields using a Zeiss Axio Imager Z2 light microscope (magnification, ×100).

### Flow cytometry

Cells were seeded in six‐well plates at a proper density and grown to approximately 80% confluence. Next, cells were harvested using trypsin without EDTA to produce a single‐cell suspension. For cell cycle analysis, suspended cells (1 × 10^6^ cells/ml) were fixed in ice‐cold 70% ethanol for 12 h, washed, and incubated in the dark with 500 ml PI/RNase staining buffer (BD Pharmingen) for 15 min at room temperature. Samples were analyzed using a BD LSRFortessa and Cell Quest Software (Becton Dickinson). All experiments were carried out at least three times in duplicate.

### Immunohistochemistry

Tissue chip analysis service was purchased from Outdo Biotech (Shanghai, China). The surgical specimens were obtained from 94 patients diagnosed with lung cancer who underwent surgery at the Department of Surgery, Peking University First Hospital, between July 2004 and June 2009. All patients were followed up until August 2014. All patients provided informed consent and confirmed that they had not received chemotherapy prior to surgery. This study was approved by the Ethics Committees of Peking University First Hospital.

Patient tumor tissues were collected, fixed in 4% paraform, embedded in paraffin, and sectioned. Sections were deparaffinized in xylene, rehydrated in graded ethanol, boiled in antigen retrieval solution for 20 min, and incubated with fresh 3% hydrogen peroxide in methanol to quench endogenous peroxidase. Next, the sections were blocked, incubated with hnRNPK monoclonal antibody (diluted to 1:100, Abcam) overnight at 4°C in a humidified chamber, and then incubated with HRP conjugated secondary antibody for 1 h. Freshly prepared diaminobenzidine (DAB) was added to the sections, and nuclei were counterstained with Mayer's hematoxylin after gentle rinsing with running water. Finally, the sections were dehydrated, covered with a coverslip, and observed under a microscope.

For the negative control, PBS was used instead of the primary antibody. Patient samples were independently scored by two pathologists who were blinded to the clinicopathological characteristics. Cases were then classified into hnRNPK high or low expression groups according to the staining intensity of hnRNPK and percentage of positive cells. Briefly, the intensity of positive cells was graded as follows: 0, negative; 1, light yellow, weak; 2, yellow brown, moderate; or 3, brown, strong. Percentage of positive cells was graded as follows: 0, no staining; 1， <10% positive cells; 2, 10%–24% positive cells; 3, 25%–49% of positive cells; 4, 50%–74% of positive cells; or 5, ≥75% of positive cells. The sum of these two individual parameters resulted in a total score ranging from 0 to 8. BRM expression level was categorized as low or high according to the mean of total score. All section slides was scanned under a microscope and photographed (magnifications: 100× and 200×). Tumor cells in five randomly selected fields were counted to determine the percentage of hnRNPK‐positive cells.

### Tumor xenograft model

Animal experiments were approved by the Biomedical Ethical Committee of the Chinese Academy of Medical Sciences (GR16002). Female BALB/c nude mice aged 6–8 weeks (Beijing Huafukang Biotechnology) were housed under specific pathogen‐free conditions. A549 cells were subcutaneously injected into each mouse (2.5 × 10^6^ suspended in 200 μl of PBS). Tumor length (L) and width (W) were measured every 3 days. Tumor volume was calculated as (W^2^L) / 2. When the tumor volume reached 50 mm^3^, hnRNPK (si‐hnRNPK) and control (si‐NC) siRNA were intratumorally injected by 3.5 nmol/40 μl of each tumor every 3 days. At 38th day, all mice were sacrificed, and solid tumors were harvested, weighed, and imaged.

Mice in another group were injected into antibody to hnRNPK (iAb‐hnRNPK) vector. The expression vector encoding intracellular iAb‐hnRNPK was generated as previous studies.^8^ When the tumor volume reached 50 mm^3^, iAb‐hnRNPK vector was intratumorally injected by 1 μg/40 μl of each tumor every 2 days. Control mice were injected with saline in the same way. On the 24th day, all mice were sacrificed, and solid tumors were harvested.

### Statistical analysis

Statistical analysis was performed using SPSS version 19.0 software (SPSS). All results are presented as mean and standard deviation (SD), unless otherwise stated. Comparisons of two groups were performed by paired or unpaired two‐tailed Student's *t*‐test. Comparisons of more than two groups were performed by one‐way ANOVA. Correlations between hnRNPK level and clinicopathological characteristics were analyzed using Chi‐square test. Kaplan–Meier method and corresponding log‐rank test were performed to assess differences in postoperative survival rates. Statistical significance is reported as *p < 0.05, **p < 0.01 or ***p < 0.001.

## RESULTS

### Expression patterns of hnRNPK differed in lung cancer tissues

**FIGURE 1:**
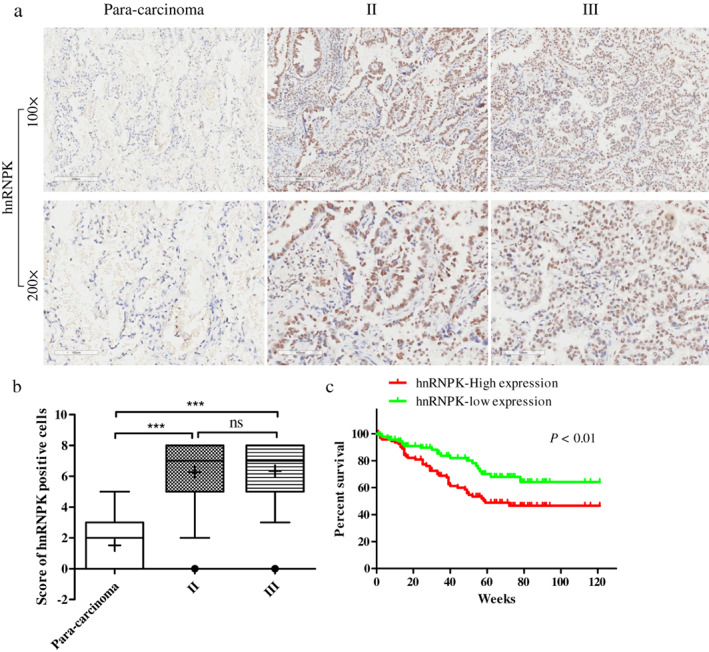
Expression patterns of hnRNPK in lung cancer tissues. (a) Immunohistochemical analysis of hnRNPK expression in 94 lung cancer samples and (b) the score of hnRNPK expression. (c) Survival curves were compared using the Kaplan–Maier method according to hnRNPK expression levels. The log‐rank test was performed to evaluate statistical significance.**p < 0.01, ***p < 0.001

To assess the clinical significance of hnRNPK, we assessed hnRNPK expression patterns in lung cancer tissues of 94 patients who had not received chemotherapy before surgery. HnRNPK levels varied greatly in lung cancer cells (Figures 1a–b). Patients were divided into hnRNPK‐high (65/94) or hnRNPK‐low groups (29/94) according to semiquantitative assessment of hnRNPK staining. Notably, hnRNPK expression was significantly correlated with tumor TNM stage (Table 1). Furthermore, the overall survival rate of the hnRNPK‐high group was significantly lower than that of the hnRNPK‐low group (Figure 1c). Collectively, these results suggest that hnRNPK expression is dynamic and that hnRNPK plays an important role in the development of lung cancer.

**TABLE 1:** Comparison of patient and tumor characteristics between tumor specimens negative or positive for hnRNPK expression (*N* = 94)

Features		Negative	Positive	*p*‐value
Age	≤50	3	8	0.7047
	>50	18	65	
TNM stage	II	17	40	0.0420*
	III	4	33	
Lymphatic invasion	Yes	1	11	0.2879
	No	20	62	
Tumor size	≤4 cm	6	33	0.2138
	>4 cm	15	40	
Malignancy grade	Il	11	34	0.8048
	III	10	39	
Gender	Male	10	43	0.4554
	Female	11	30	
Distant metastasis	Yes	3	7	0.6878
	No	18	66	

*Note*: Italic value in Table 1 indicates *p* < 0.05 and hnRNPK expression was significantly correlated with tumor TNM stage.

### hnRNPK knockdown inhibited lung cancer cell proliferation and migration in vitro

**FIGURE 2:**
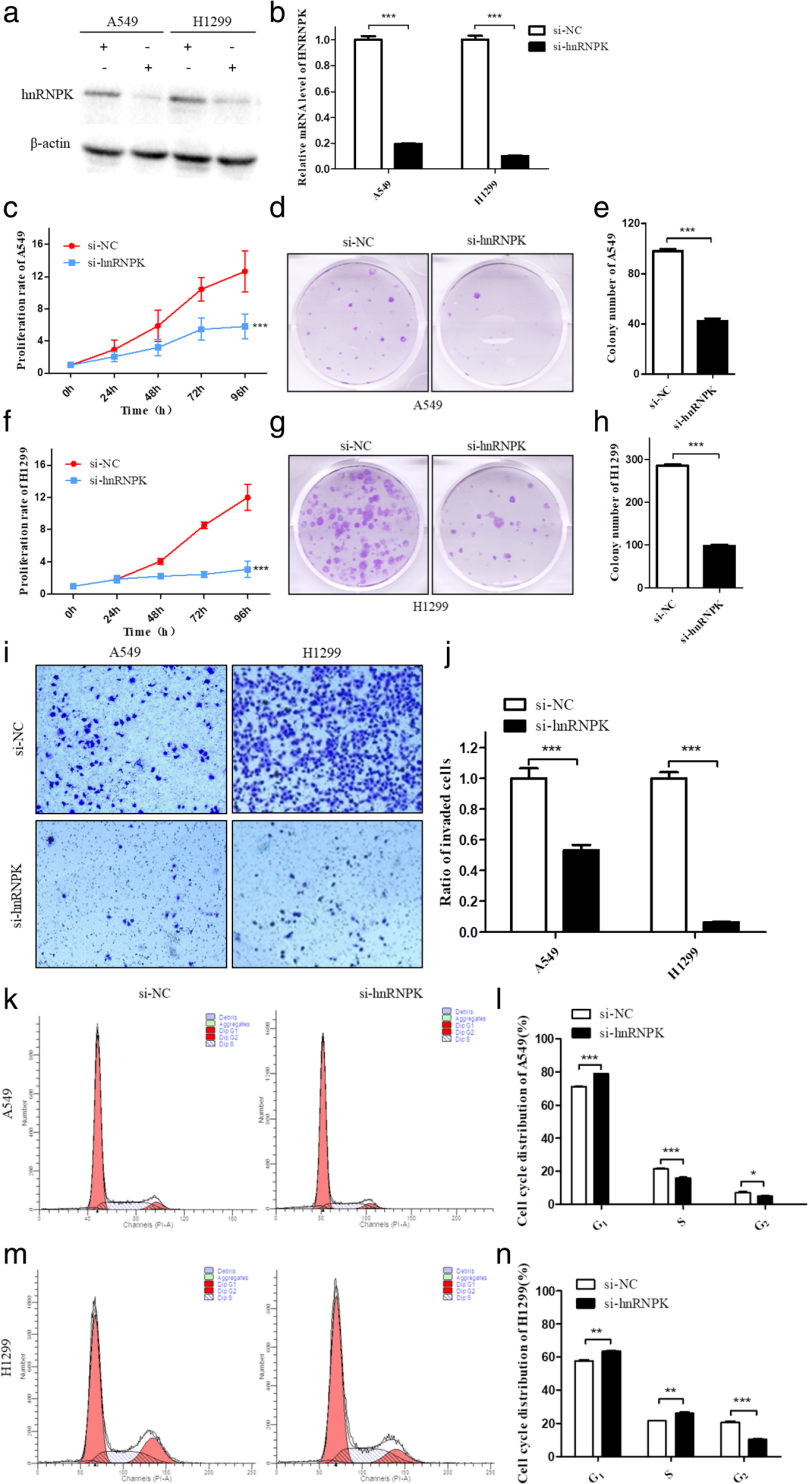
hnRNPK knockdown affected lung cancer cell proliferation, migration and cell cycle progression. (a) Expression of hnRNPK protein in hnRNPK‐knockdown cell lines and negative controls was detected by western blotting. β‐actin was used as a loading control. (b) Expression of hnRNPK mRNA in hnRNPK‐knockdown cell lines and negative controls was detected by PCR. GAPDH was used as an endogenous control. (c–h) Lung cancer cell proliferation was measured by CCK‐8 and colony formation assays. (i–j) Lung cancer cell migration was measured by transwell assay. (k–n) Flow cytometry analysis of cell cycle distribution in each cell line. *p < 0.05, **p < 0.01, ***p < 0.001

Considering high hnRNPK expression in lung cancer tissues, we transfected siRNA with a specific hnRNPK knockdown into the human lung cancer cell lines A549 and H1299. hnRNPK expression was confirmed by western blotting (Figure 2a) and fluorescent real‐time quantitative PCR (Figure 2b). CCK‐8 assay and colony forming assay revealed that the cell proliferation rate of hnRNPK knockdown was significantly lower than that of control cells (Figures 2c–h). These results suggest that hnRNPK promotes lung cancer cell proliferation. In addition, the hnRNPK knockdown significantly inhibited the migratory ability of A549 and H1299 cells (Figure 2i‐j). Importantly, following simultaneous results revealed, hnRNPK‐knockdown A549 cells were arrested at the G_1_/S phase (Figure 2k‐l). The H1299 cells, however, were slightly differently (Figure 2m‐n). Together, these observations suggest that hnRNPK acts as a promoting factor in lung cancer cells by regulating cell cycle.

### hnRNPK knockdown effectively inhibited the progression of lung cancer in vivo

**FIGURE 3:**
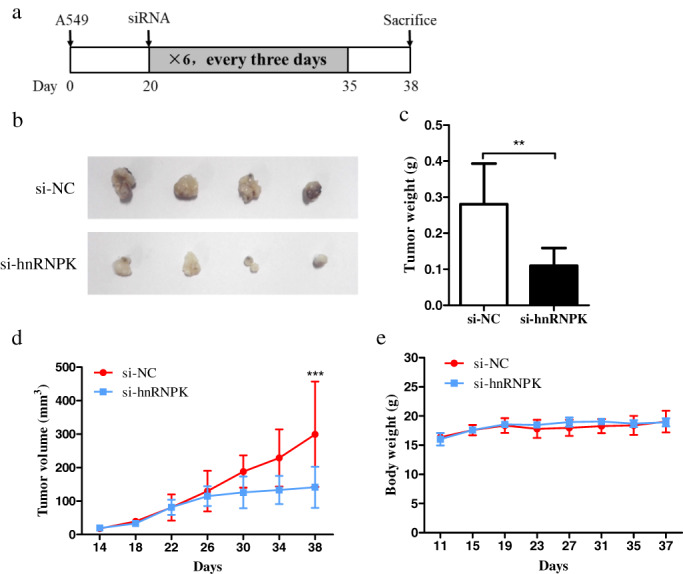
hnRNPK knockdown by siRNA suppresses the growth of A549 xenograft tumors in vivo. (a) Schematic of the mouse xenograft model. (b) Size of tumors derived from si‐hnRNPK or si‐NC in four female nude mice/group are presented. (c) Tumor weight of the xenografts from the two groups. (d) Tumor volume of xenografts derived from the two groups were measured every 3 days and are presented as growth curves. (e) Bodyweight of the mice in the two groups at the indicated time‐points. Error bars represent the mean ± SD of three independent experiments. **p < 0.01, ***p < 0.001

**FIGURE 4:**
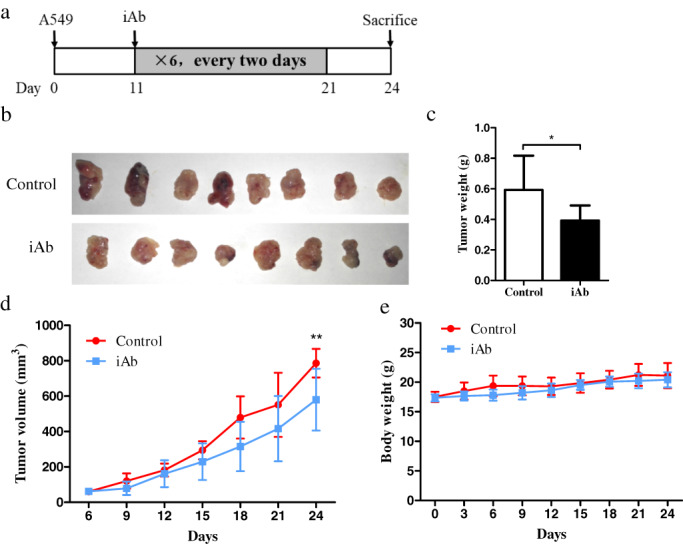
hnRNPK knockdown by iAb vector suppresses the growth of A549 xenograft tumors in vivo. (a) Schematic of the mouse xenograft model. (b) Size of tumors derived from iAb‐hnRNPK or negative control in four female nude mice/group are presented. (c) Tumor weight of the xenografts from the two groups. (d) Tumor volume of xenografts derived from the two groups were measured every 3 days and are presented as growth curves. (e) Bodyweight of the mice in the two groups at the indicated time‐points. Error bars represent the mean ± SD of three independent experiments. *p < 0.05, **p < 0.01

Next, we established a xenograft mouse model by subcutaneously injection with A549 cells. The mice were divided into two groups. In the first group, when the tumor volume reached 50mm^3^, siRNA‐hnRNPK and siRNA‐NC were intratumorally injected, respectively every three days(Figure 3a). In the second group, when the tumor volume reached 50mm^3^, iAb‐hnRNPK vector and saline were intratumorally injected respectively every two days (Figure 4a). At the end of the experiment, the mean tumor volume and weight of the hnRNPK‐knockdown group was significantly smaller than that of the control group. The overall effect of hnRNPK knockdown on tumor growth was statistically significant (Figures 3b–d and Figures 4b–d). While there were no significant differences recorded in bodyweight of mice between the two groups((Figure 3e and Figure 4e). These results of in vivo experiments support our hypothesis that hnRNPK is critical for the proliferation and survival of lung cancer cells.

### hnRNPK inhibited the p53‐dependent signaling pathway in lung cancer

**FIGURE 5:**
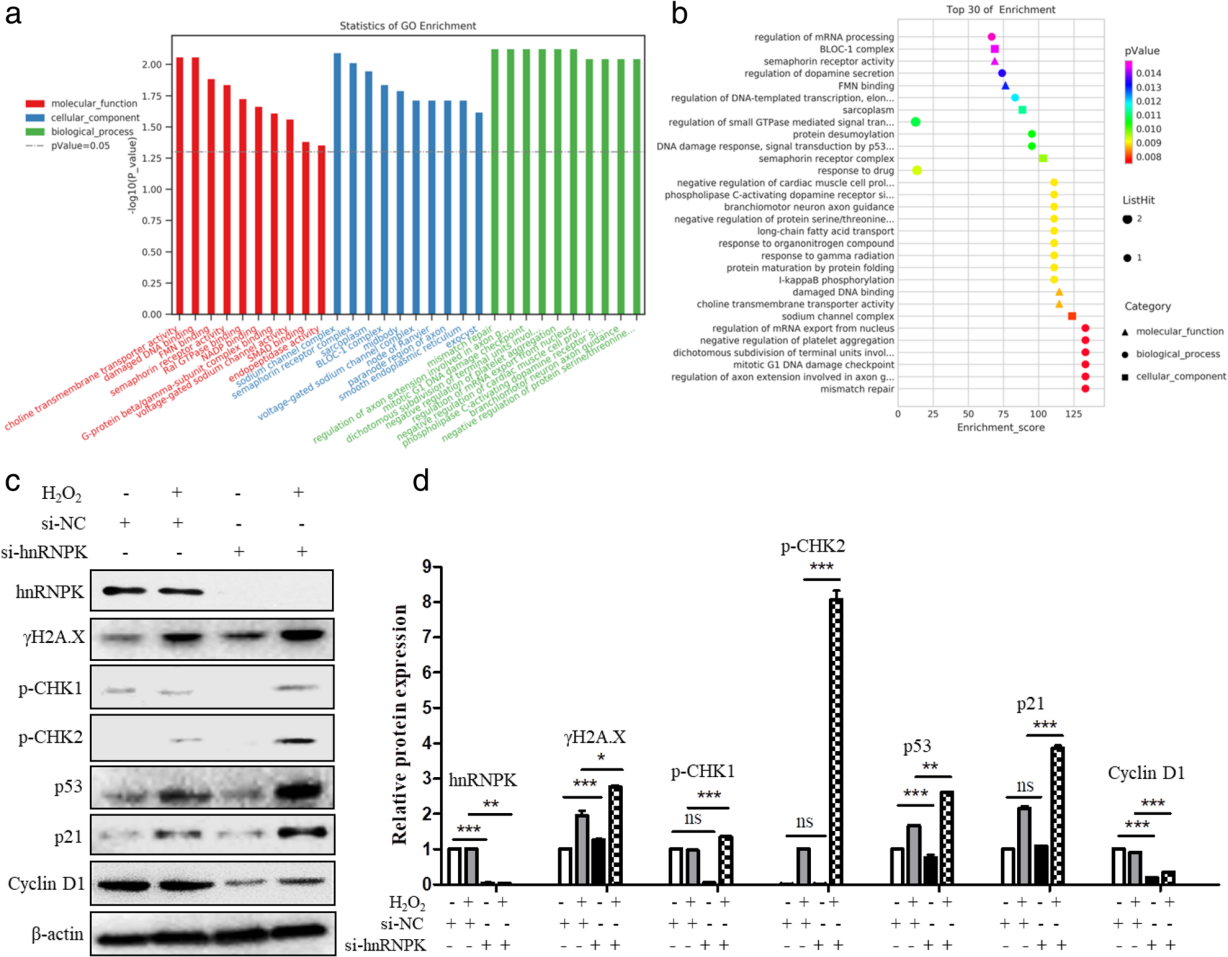
GO functional term and KEGG signaling pathway enrichment analyses showed that hnRNPK knockdown activated the p53‐dependent signaling pathway. (a) GO annotation of downregulated mRNAs with the top 10 enrichment scores in the categories of molecular functions, cellular components and biological process. (b) KEGG signaling pathway enrichment analysis of downregulated mRNAs with the top 30 enrichment scores. (c–d) Western blotting detected the protein expression of associated signaling pathway after DNA damage and hnRNPK knockdown in A549 cells. *p < 0.05, **p < 0.01, ***p < 0.001

To determine the molecular mechanisms underlying the phenotypic changes in hnRNPK‐knockdown A549 cells, mRNA microarray analysis was performed. GO functional term enrichment analysis of the significantly differentially expressed genes of hnRNPK knockdown cells compared with the negative control cells was used to identify the role of differentially expressed hnRNPK in Figure 5a. KEGG signaling pathway enrichment analysis of the significantly differentially expressed genes was used to elucidate the pathways and molecular interactions of hnRNPK in A549. The top 30 pathways associated with the downregulated mRNAs are listed in Figure 5b. Among these 30 pathways by hnRNPK‐knockdown, “DNA damage response，signal transduction by p53”,“damage DNA binding” and “mitotic G1 DNA damage checkpoint” were similar to some signaling pathways in Figure 5a. These signaling pathways were identified and which implied that the p53 signaling pathway may be a target for hnRNPK as shown in our previous studies.^9^ Chen et al.^8^ have reported the antiapoptotic activity of hnRNPK through regulation of downstream antiapoptotic genes in nasopharyngeal carcinoma, indicating that hnRNPK binds to the promoter of the antiapoptotic gene FLIP to induce its transcription. HnRNPK deletion can result in transcriptional inactivation of the p53 target gene, leading to defects in cell cycle due to DNA damage in human colon carcinoma cell line HCT‐116. DNA damage induces hnRNPK modification via small ubiquitin‐like modifier proteins, which regulate the transcriptional activation of p53.^10^ Therefore, we hypothesized that the carcinogenesis‐promoting effects of hnRNPK in lung cancer are regulated by the inhibition of the p53–dependent signaling pathway.

In flow cytometry and cell cycle accumulation mapping, the G_1_ phase was significantly prolonged (Figures 2k–l). The inhibition of cell proliferation was most likely mediated by cell cycle arrest. The results of p53 activation indicated cell cycle arrest following hnRNPK knockdown. DNA integrity is assessed at the G_1_/S checkpoint, and the cell cycle can arrest at this point in response to incorrectly or partially replicated DNA.^11^ As mentioned above, previous studies have shown that hnRNPK and p53 are synergistically involved in the regulation of DNA damage repair and that hnRNPK can enhance its affinity to p53 and regulate downstream signaling pathways through its methylation, lysine ubiquitination, and silk/threonine phosphorylation.^12^ Thus, we hypothesized that, in A549 cells with stable hnRNPK knockdown, p53 was activated through DDR induction.

**FIGURE 6:**
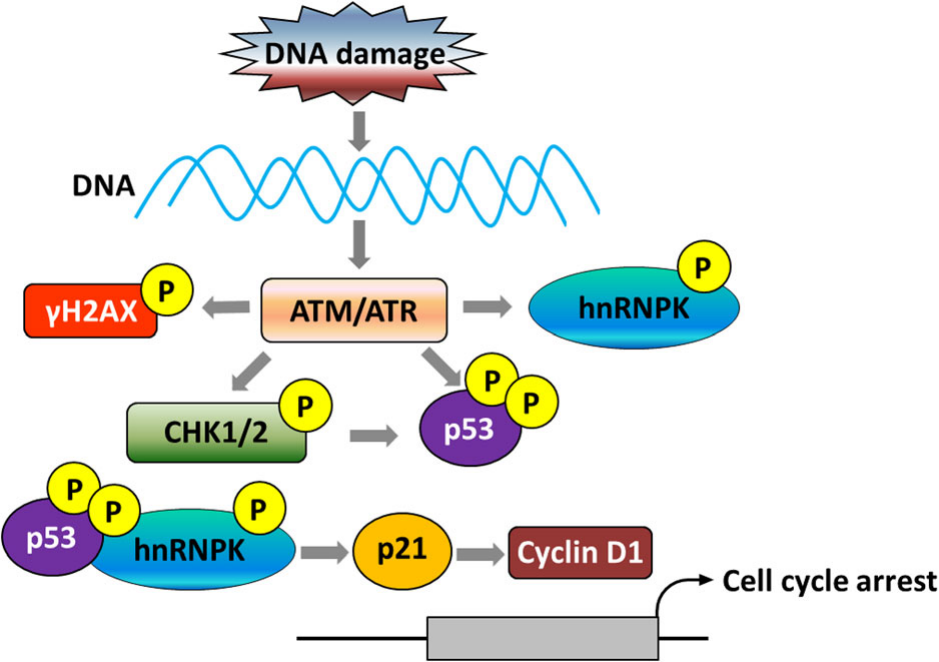
Inhibition of progression of lung cancer cells induced by hnRNPK‐knockdown is dependent on activation of p53 by the p53/p21/cyclin D1 pathway. In response to DNA damage, ATM and ATR are activated and then phosphorylate H2A.X. The signaling transducers CHK1 and CHK2 are also phosphorylated subsequently leading to the phosphorylation and stabilization of hnRNPK and p53. This can transcriptionally activate the downstream signals such as p21 and other DNA damage repair associated genes. Therefore, p21 interacts with cyclin D1 finally resulting in cell cycle arrest

To confirm this hypothesis, we induced DNA damage of hnRNPK‐knockdown A549 cells by H_2_O_2_ and examined related signaling pathways expression (Figures 5c–d). We examined the expression of γH2A.X of DNA damage using western blotting. The expression level of γH2A.X in A549 cells with stable hnRNPK knockdown was significantly higher than that in control cells. γH2A.X at serine 139 is an early landmark event following the induction of DNA double‐strand breaks (DSBs).^13^ γH2A.X recruits repair factors to the nucleus following DNA damage.^14^ When DNA DSBs are induced, ATM and ATR are activated and phosphorylates a range of target molecules, specifically CHK1/2 and p53.^15^ ATM and ATR are important protein kinase and can respond to DNA damage and arrest cell cycle at the G_1_/S stage. CHK1 is another protein kinase with a function similar to that of CHK2 and has stringent signal transduction functions in cell cycle regulation and response detection sites.^16^ In mammalian cells, DSB signals detected by ATM are transduced by CHK2, and UV damage signals detected by ATR are transduced by CHK1.^17^ However, functions of proteins in these two pathways may overlap to some extent. In A549 cells with stable hnRNPK knockdown, altered phosphorylation levels of CHK1/2 were observed, while p53, p21 and Cyclin D1 were significantly activated. These results clearly indicate when DNA damage occurs, low hnRNPK expression can initiate DDR via the ATM/ATR/CHK1/2 pathway, thereby activating p53 and associated downstream signaling pathway (Figure 6) .

We simultaneously detected a difference in protein expression between the normal H1299 cell line and the H1299 cell line with stable hnRNPK knockdown using western blotting (Figures S1a‐b). The results showed that there was no p53 expression in H1299 cells. Futhermore, there was a significant difference in other proteins expression between H1299 and A549 cells with hnRNPK–knockdown. H1299 is the cell line lacking p53. It can be hypothesized that hnRNPK knockdown may inhibit H1299 cells proliferation and migration via other signaling pathways. Thus hnRNPK‐knockdown H1299 cells could not be arrested at the G1/S phase (Figures 2m–n). To what extent hnRNPK is able to interact with p53‐lost cell lines are not fully understood, and remains to be confirmed in further experiments.

## DISCUSSION

hnRNPK is a member of the hnRNP family, exhibits RNA and DNA linkage sites and is involved in several cellular processes, including gene regulation and signal transduction. The development and progression of various human tumors are associated with the action of hnRNPs.^18^, ^19^, ^20^ However, research on this family is still in its infancy, and many key mechanisms remain unclear. Therefore, molecular mechanisms underlying the effects of hnRNPK warrant further elucidation.

Recent studies have shown that hnRNPK is highly expressed in many human tumor cells, which is consistent with our previous findings that it plays an important role in tumorigenesis. Moreover, in our previous studies, we demonstrated that hnRNPK expression is strongly correlated with biological processes such as cell migration and invasion and that it is an important factor for lung cancer metastasis.^21^ hnRNPK overexpression in mouse fibroblasts (NIH3T3) resulted in increased malignancy, cell migration and infiltration, and tumorigenicity of NIH3T3 cells.^8^ Similarly, other studies have revealed that hnRNPK was highly expressed in primary lung cancer tissues and the positive rate of hnRNPK expression was higher in cancer tissue that in normal tissues and inflammatory controls. These findings suggest that hnRNPK plays an important role in the development of lung cancer. However, the molecular mechanism underlying its role in the development and progression of lung cancer remains unclear.^22^

hnRNPK plays roles in the regulation of expression of various oncogenes and tumor suppressor genes, promotion of cell proliferation and division, and development of various tumors. Ostareck‐Lederer et al. have demonstrated that hnRNPK activates c‐Src, which in turn phosphorylates the tyrosine at the position 458 of the KH3 domain of hnRNPK, thereby inactivating and inhibiting cytosolic hnRNPK protein components. Subsequently, it forms a complex with DICE to initiate translation.^23^ Furthermore, hnRNPK affects c‐Myc activity at both transcriptional and translational levels and promotes c‐Myc transcription by binding to the pyrimidine‐rich region (CT element) of the c‐Myc promoter both in vivo and in vitro.^24^ High hnRNPK expression in breast cancer cells, prostate cancer cells, and melanoma tissues is reportedly accompanied by elevated c‐Myc levels.^2^, ^25^, ^26^ In hepatocarcinoma tissues and cells, Tcl1 activates G6PD in an hnRNPK‐dependent manner and promotes the processing of G6PD precursor RNA and its protein expression. Conversely, the tumor suppressor gene encoding protein PTEN forms a complex with hnRNPK to inhibit its activity, and shearing of G6PD precursor RNA inhibits liver cancer. As a transcription factor, hnRNPK binds to the eIF4E promoter region and 3′ untranslated region of p21 mRNA, inhibiting p21 translation and increasing translation initiation, cell division, and tumor formation.^27^, ^28^ Liu et al.^29^have demonstrated that hnRNPK may serve as a candidate diagnostic biomarker and a promising therapeutic target for head and neck squamous cell carcinoma (HNSCC). The Wnt/β‐catenin signaling pathway may be a possible downstream signaling pathway of hnRNPK for HNSCC. Knockdown of hnRNPK significantly decreased HNSCC proliferation and migration, and downregulated the expression levels of Wnt/β‐catenin signaling pathway‐related proteins. Thus, alterations in hnRNPK levels or its post‐transcriptional modifications can modulate several key pathways in tumorigenesis. Despite these findings, it is challenging to determine whether hnRNPK can be used as a driving gene of tumorigenesis. The role of hnRNPK in tumor development likely depends on tissue type and microenvironment, including recruitment of bound RNA, DNA, and proteins. hnRNPK is highly expressed in most tumors, and its expression is negatively correlated with prognosis; however, this lack of tissue specificity has limited potential applications of hnRNPK as a diagnostic marker for tumors. A hnRNPK knockout mouse model demonstrated that hnRNPK deletion was associated with mouse growth and development, whereas complete deletion led to embryonic lethality. Moreover, hnRNPK induced defects in growth and development of mice, resulting in their increased susceptibility to hematological malignancies and lymphoma. These findings indicate that hnRNPK may function as a tumor suppressor gene in hematological malignancies and lymphomas. Development of a transgenic mouse model expressing wild‐type hnRNPK is warranted for exploring the effects of hnRNPK overexpression on tumorigenesis and development, which can further aid the development of hnRNPK overexpression‐dependent targeted drugs.

Lung cancer is divided into small cell lung cancer and non‐small cell lung cancer (adenocarcinoma, squamous cell carcinoma and large cell lung cancer) according to the pathological type. Non‐small cell lung cancer (NSCLC) accounts for 80%–85% of lung cancer, and the 5‐year survival rate is very low.^30^, ^31^ A549 and H1299 cells are epithelial cells and belong to human NSCLC cell line. Both cells proliferate quickly under normal conditions (Figures 2c–h). The difference is that A549 cell line is directly derived from the patient's lung carcinomatous tissue, whereas the H1299 cell line is derived from the patient's lymph node metastasis of the lung. The H1299 cell line has a homozygous partial deletion of the p53 protein, and lacks expression of p53 protein.^32^ In this study, A549 and H1299 cells were used as materials to study the functions of hnRNKP in the progression of NSCLC. Our previous studies have shown that hnRNPK involves in the progression of gastric cancer via p53/p21/CCND1 pathway.^9^ It is hypothesized that hnRNPK also plays a role via the similar signaling pathway in lung cancer. In order to study whether hnRNPK plays a role through the same mechanism in the absence of p53, we used H1299 cells as a control. Our study confirmed that hnRNPK knockdown inhibited proliferation and migration of A549 and H1299 cells in vitro. Moreover, hnRNPK‐knockdown A549 cells were arrested at the G1/S phase (Figures 2k–l) and the p53‐dependent signaling pathway was inhibited in A549 cells (Figures 5c–d). However, the protein expression changes of related signaling pathway were different in hnRNPK‐knockdown H1299 cells (Figure S1). It can be hypothesized that hnRNPK knockdown may inhibit H1299 cells proliferation and migration via other signaling pathways.

In summary, we found that hnRNPK knockdown significantly inhibited the proliferation and migration of lung cancer cells. In addition, hnRNPK knockdown elevated DNA damage levels and led to the upregulation of the protein expression levels of pCHK1, pCHK2, p53, p21 and cyclin D1 in A549 cells. These results confirmed that hnRNPK may promote the progression of lung cancer by inhibiting the p53‐dependent signaling pathway. HnRNPK could therefore be a potential therapeutic target for the treatment of lung cancer.

## CONFLICT OF INTEREST

The authors declare that they have no competing interests.

## Supporting information


Figure S1

Table S1
