# Supplementary figures and images for: Heterogeneous nuclear ribonucleoprotein K promotes the progression of lung cancer by inhibiting the p53‐dependent signaling pathway
Source: Thorac Cancer. 2022 Mar 29;13(9):1311–21. doi: 10.1111/1759-7714.14387 (PMC9058298; doi:10.1111/1759-7714.14387)

## Slide 1
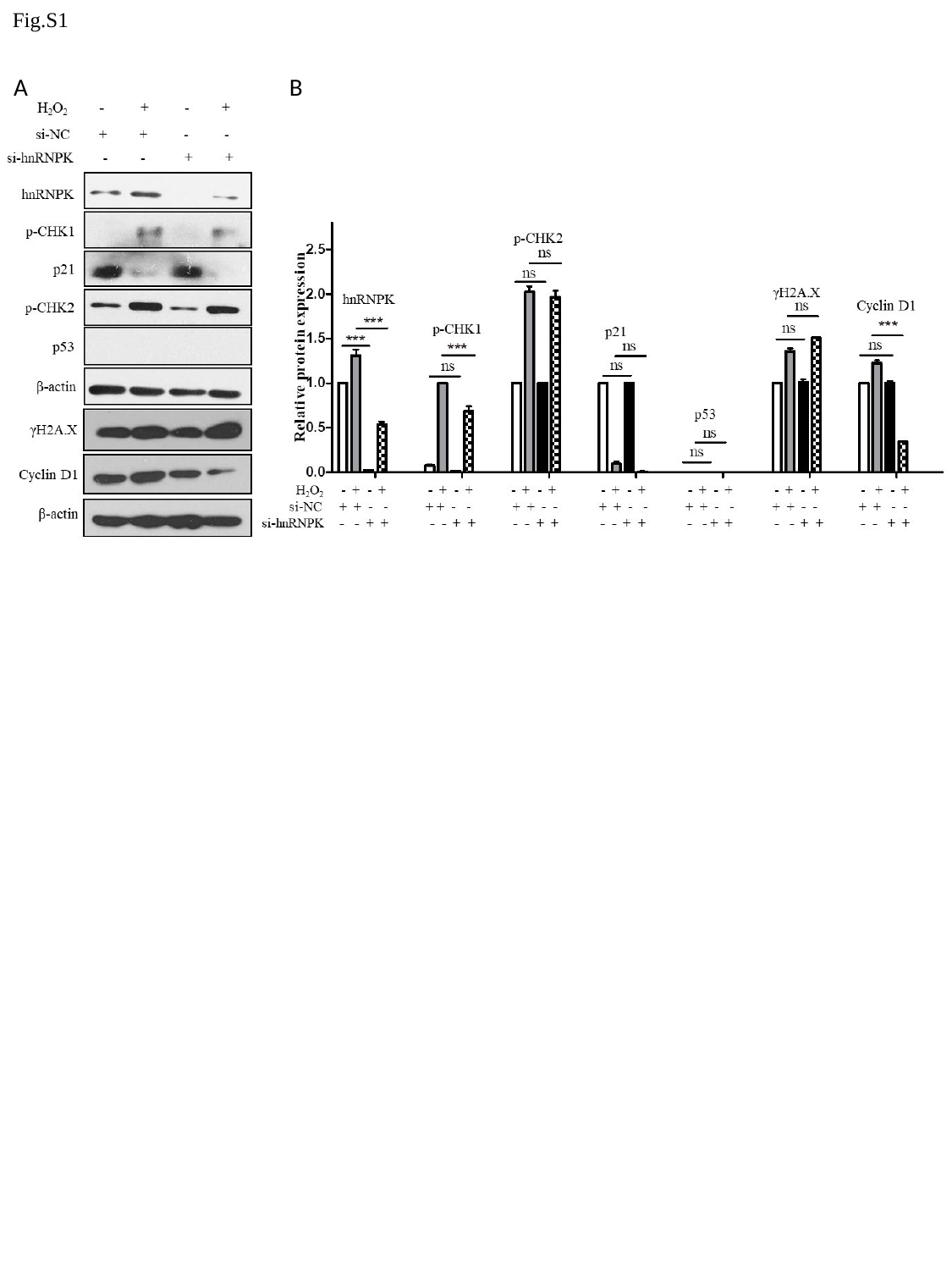

Fig.S1
A
B

Supplement: Supplementary file 1 — Figure S1 [file TCA-13-1311-s002.pptx]
