# Supplementary material for: Heterogeneous nuclear ribonucleoprotein K promotes the progression of lung cancer by inhibiting the p53‐dependent signaling pathway
Source: Thorac Cancer. 2022 Mar 29;13(9):1311–21. doi: 10.1111/1759-7714.14387 (PMC9058298; doi:10.1111/1759-7714.14387)
Supplement: Supplementary file 2 — Table S1 [file TCA-13-1311-s001.docx]

Table S1 Primer information

| Gene |  | Primer sequence (5’-3’) |
| --- | --- | --- |
| GAPDH | Forward: | ACGGATTTGG TCGTATTGGG |
|  | Reverse | TGATTTTGGAGGGATCTCGC |
| hnRNPK | Forward: | AGACCTGGAGACCGTTAC |
|  | Reverse | ATAAGCCATCTGCCATTC |
